# The 19th Rocky Mountain Virology Association Meeting

**DOI:** 10.3390/v12010085

**Published:** 2020-01-11

**Authors:** Joel Rovnak, Laura A. St. Clair, Elena Lian, Carley McAlister, Rushika Perera, Randall J. Cohrs

**Affiliations:** 1 Department of Microbiology, Immunology and Pathology, Colorado State University, Fort Collins, CO 80523, USA; Joel.Rovnak@colostate.edu; 2 Arthropod-borne and Infectious Diseases Laboratory, Department of Microbiology, Immunology and Pathology, Colorado State University, Fort Collins, CO 80523, USA; stclairl@colostate.edu (L.A.S.C.); elian@rams.colostate.edu (E.L.); carleym@rams.colostate.edu (C.M.); Rushika.perera@colostate.edu (R.P.); 3 Departments of Neurology and Immunology/Microbiology, University of Colorado School of Medicine, Aurora, CO 80045, USA

**Keywords:** virus, prion, interferon, flavivirus, retrovirus, epidemiology, RNA polymerase II, transmission, herpesvirus, host-virus interaction

## Abstract

This autumn, 95 scientists and students from the Rocky Mountain area, along with invited speakers from Colorado, California, Montana, Florida, Louisiana, New York, Maryland, and India, attended the 19th annual meeting of the Rocky Mountain Virology Association that was held at the Colorado State University Mountain Campus located in the Rocky Mountains. The two-day gathering featured 30 talks and 13 posters—all of which focused on specific areas of current virology and prion protein research. The keynote presentation reviewed new tools for microbial discovery and diagnostics. This timely discussion described the opportunities new investigators have to expand the field of microbiology into chronic and acute diseases, the pitfalls of sensitive molecular methods for pathogen discovery, and ways in which microbiology help us understand disruptions in the social fabric that pose pandemic threats at least as real as Ebola or influenza. Other areas of interest included host factors that influence virus replication, in-depth analysis of virus transcription and its effect on host gene expression, and multiple discussions of virus pathology, epidemiology as well as new avenues of diagnosis and treatment. The meeting was held at the peak of fall Aspen colors, surrounded by five mountains >11,000 ft (3.3 km), where the secluded campus provided the ideal setting for extended discussions, outdoor exercise and stargazing. On behalf of the Rocky Mountain Virology Association, this report summarizes 43 selected presentations.

## 1. Introduction

**Figure 1:**
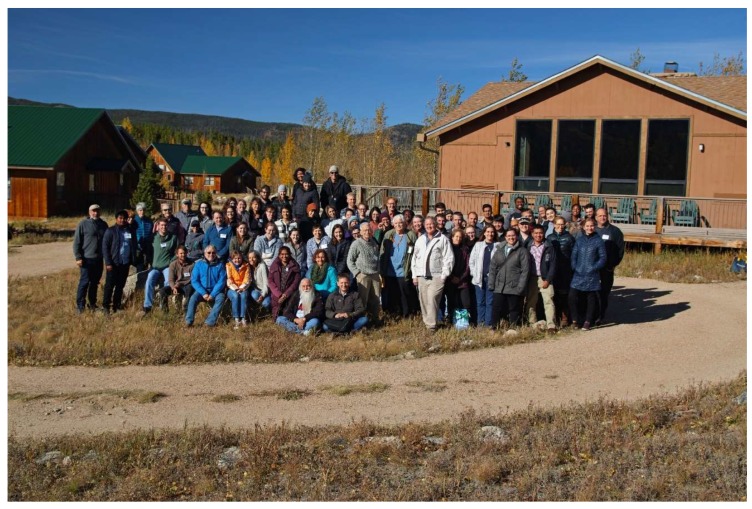
Attendees at the 19th annual Rocky Mountain Virology Association meeting.

**Figure 2:**
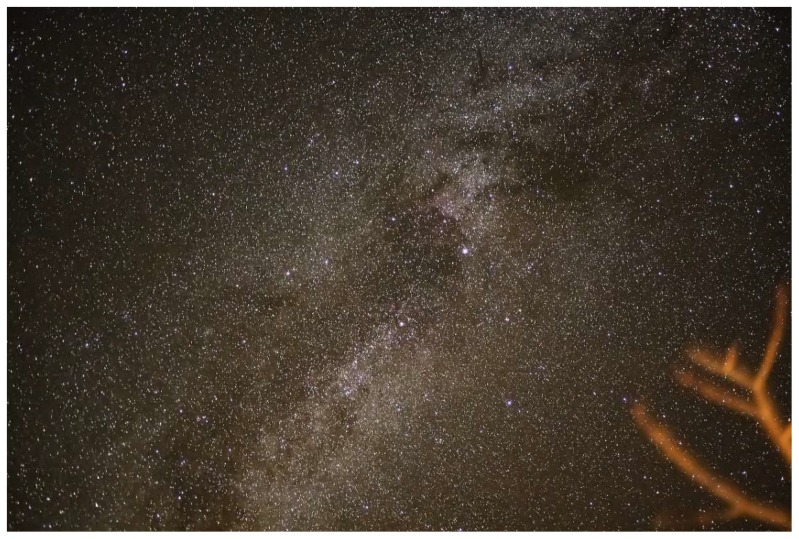
Night skies at 19th Rocky Mountain Virology Association meeting. Picture: exposure: 20 s; focal length: 40 mm; aperture: f/1.7; ISO: 3200; credit, Dr. Joseph Timpona, University of Colorado Boulder.

The Rocky Mountain Virology Association was established in 2000 to address the critical need of a venue to showcase regional scientists investigating all facets of host–virus interactions in order to provide an environment where graduate students could interact with more established virologists, and new investigators could establish productive collaborations. The Colorado State University Mountain Campus, located 50 miles west of Fort Collins, Colorado, was selected as the venue since its location at the base of the continental divide provided sufficient seclusion to enhance attendance at all presentations and programed free time was provided to explore the beautiful mountain setting. Through the years, the meeting has grown to include significant contributions from prion biologists. With many national and international speakers, the meeting has grown past its regional focus. Nonetheless, the annual RMVA meeting has maintained its unique ability to provide a forum to advance science and mentorship. Professional childcare was again provided to facilitate attendance by individuals with young children. During the poster session, the kids preformed “Kidtagion,” a skit that portrayed pathogen transmission through fomites. The production obtained glowing reviews from the keynote speaker, Dr. W. Ian Lipkin (a.k.a. The Virus Hunter), who was also the chief scientific advisor of the 2011 film *Contagion*. Taken together, the 19th annual Rocky Mountain Virology Association meeting (Figure 1) continued its legacy of virology, prion biology, and mentorship and at an altitude of 9000 ft (2.7 km). With clear night skies, it was easy to see that not all the stars were at the podium (Figure 2). Selected abstracts are presented below.

## 2. Summary of Scientific Sessions

Dr. Jayasri Das Sarma (Indian Institute of Science Education and Research Kolkata, Kolkata, India) opened the meeting with her invited presentation describing the protective role of Ifit2 during neurotropic virus infection. Dr. W. Ian Lipkin followed with his keynote talk describing new technologies used to identify and analyze novel pathogens. Immediately afterwards, Dr. Lipkin fielded questions from students and early investigators at the annual speaker’s fireside chat. During the next two days, the focus of the meeting enlarged to include multiple host factors induced following virus infection, results from next-generation analysis of host and virus transcription, and in-depth presentations describing virus pathology, epidemiology. Invited speakers described pathogen transmission from bats to humans (Dr. Raina Plowright, Montana State University, Bozeman, MT, USA) and among cats (Dr. Sue VandeWoude, Colorado State University, Fort Collins, CO, USA). Dr Sabra Klein, Johns Hopkins Bloomberg School of Public Health, Baltimore, MD, USA, described how biological sex effects adaptive immune responses to pathogens and vaccines. Drs. David Bloom (University of Florida, Gainesville, FL, USA), Joel Baines (Louisiana State University, Baton Rouge, LA, USA) and Sven Heinz (University of California, San Diego, CA, USA) provided a series of presentations that provided an in-depth look at virus-encoded non-coding RNA during latency and reactivation, the precise positioning of RNA polymerase II on the virus genome during productive infection, and how a virus-encoded protein alters host transcription termination leading to altered chromatin configurations. New areas of pathogen diagnosis and treatment provided uplifting outlooks on the future; an excellent ending to an exciting meeting. Following is a synopsis of selected presentations.

### 2.1. Host Factors

Rebecca Broeckel, along with Emily Fitzmeyer and Sonja Best (Rocky Mountain Laboratories, NIAID, NIH, and Hamilton, MT, USA), investigated the restriction of tick-borne flaviviruses by tripartite motif containing protein 5α. Tick-borne and mosquito-borne flaviviruses (TBFVs and MBFVs) cause fatal hemorrhagic fevers or neurological disease in humans. Type I interferon (IFN) is critical for host resistance to flaviviruses, but its mechanism of action and potential for specific recognition of different flavivirus species is not fully understood. Tripartite motif containing protein 5a (*TRIM5a*) is an IFN-stimulated gene originally identified as a major restriction factor for HIV-1 in rhesus macaques. Their group recently discovered that TRIM5a from both humans and non-human primates significantly impairs replication of TBFVs, but not MBFVs. However, the precise flavivirus molecular target of *TRIM5a* is not known. To determine the mechanism of *TRIM5a* restriction, a sensitive TBFV called Langat virus (LGTV) was passaged in cells stably overexpressing *TRIM5a* until resistance occurred. Two independent viral passages resulted in mutations at the same valine 559 in Nonstructural protein 3 (*NS3*), the viral protease/helicase. Recombinant LGTV bearing the mutations at V559 were resistant to *TRIM5a* restriction, while the recombinant wild type LGTV remained sensitive. They showed, using electron microscopy, that Apex-tagged *TRIM5a* was recruited to the ER surrounding sites of LGTV replication complexes. Importantly, recombinant LGTV with the resistant mutations at V559 did not recruit *TRIM5a*-Apex to replication complexes, demonstrating that NS3 V559 is required for recognition by *TRIM5α*. This work is uncovering mechanisms of specificity to the IFN response in recognition and disruption of flavivirus replication complexes. The information gained will be used to determine whether escape of MBFVs from *TRIM5a* is required for infection and pathogenesis in humans. No animal or human studies were performed.

Abhilash Chiramel (Rocky Mountain Labs, NIH, Hamilton, MT, USA) investigated the role of the protein Family with Sequence Similarity 134, Member B, Transcript Variant X1 (FAM134B) in flavivirus replication. The endoplasmic reticulum (ER) forms a multipart network of continuous sheets and tubules, extending from the nuclear envelope to the plasma membrane. ER-resident protein FAM134B, serves as a key receptor for lysosomal degradation of ER (termed ER-phagy). While FAM134B depletion leads to ER expansion, FAM134B over-expression results in ER fragmentation and autolysosomal degradation, thereby controlling ER morphology. Since flavivirus replication occurs on ER membranes, they investigated the impact of ER-phagy on Zika virus (ZIKV), West Nile virus (WNV) and Dengue virus (DENV). Infection of FAM134B/ER-phagy-deficient mouse embryonic fibroblasts with flaviviruses rendered these cells highly permissive for virus replication. They found that flaviviruses utilize the viral protease (NS2B/3) to cleave both human and mouse FAM134B, potentially to facilitate ER expansion for virus replication. Importantly, they could confirm cleavage of endogenous human FAM134B upon DENV infection. Lastly, to determine the role of ER-phagy in flavivirus pathogenesis, mice deficient for *FAM134B* were infected with ZIKV. While *FAM134B* depletion in cell culture supported higher levels of flavivirus replication, *FAM134B−/−* mice were not more susceptible to ZIKV infection compared to wild-type mice. These data suggest that ER-expansion induced by flaviviruses through FAM134B cleavage may occur locally to facilitate optimal replication. However, further loss of FAM134B does not augment virus replication in vivo and is not more generally involved in modulating host immunity. These results identify a cellular target of flavivirus antagonism to promote replication and suggest that additional ER-phagy receptors (e.g., SEC62 and CCPG1) should be examined to fully elucidate the antiviral potential of ER-phagy. All animal experiments were performed as per approved protocols by the RML Animal Care and Use Committee (ACUC).

Kelly Du Pont (Department of Chemistry, Colorado State University, Fort Collins, CO, USA) presented her work investigating the role of Motif V in flavivirus nonstructural protein 3 (NS3). The unwinding of double-stranded RNA intermediates is critical for the replication and packaging of flavivirus RNA genomes. The unwinding of flavivirus dsRNA is achieved by the C-terminal helicase domain of NS3. NS3 is known to translocate along and unwind dsRNA in an ATP-dependent manner. However, the mechanism of energy transduction between the ATP and RNA binding pockets is not well understood. If we can understand how energy travels through the helicase, then we can target specific areas of the helicase for antiviral development. Previous molecular dynamics (MD) simulations published by their group have identified the conserved Motif V in NS3 is a potential “communication hub” for this energy transduction pathway. To investigate the role of Motif V, they used a combined study of molecular dynamics, biochemistry and virology to clarify how the energy of ATP hydrolysis is used to drive NS3 helicase activity. Wild-type NS3h and several mutants were identified and tested in several biochemical assays, viral replication assays, and analyzed in MD simulation. They observed that Ser411Ala possesses a hyper-active helicase phenotype, meaning the mutation causes the helicase to unwind dsRNA more quickly than wild type (WT). Interestingly, a flavivirus replicon with a Ser411Ala mutation demonstrated reduced viral replication compared to WT, suggesting that hyperactive helicase function is detrimental to viral genome replication. Overall, these data help define the linkage between ATP hydrolysis and helicase activity within NS3 through conserved Motif V and provide valuable new insight into the biophysical mechanisms for ATPase driven NS3 helicase function. No human or animal studies were performed.

Alison Gilchrist (Department of Molecular, Cellular and Developmental Biology, BioFrontiers Institute, University of Colorado Boulder, Boulder, CO, USA) investigated diacylglycerol O-acyltransferase 2 (DGAT2), a host protein that is cleaved by dengue virus during infection. Dengue virus is a mosquito-borne pathogen that is highly prevalent in tropical regions of the world and is poised to become more important to human health in temperate regions as global warming produces a warmer, wetter climate. The dengue virus genome encodes a protease which is known to cleave at least one host protein as well as the viral polypeptide. They used machine learning to predict new host targets of the dengue virus protease, in order to find undiscovered host–virus interactions during dengue virus infection. They identified that the host protein DGAT2 is recognized and cleaved by the dengue virus protease. They also identified the cleavage motif, and showed that when the cleavage motif is mutated, the dengue protease is incapable of cleaving DGAT2. They predict that dengue virus is manipulating the host lipid environment by preventing the production of triacylglycerol from diacylglycerol, a process facilitated by DGAT2. No human or animal studies were performed.

Charles Grose (University of Iowa, Iowa City, IA, USA) discussed the Inhibition of varicella-zoster virus (VZV) assembly by three divergent inhibitors of autophagy. In published papers, his laboratory has shown that VZV induces autophagy in infected cells. This increase is far above levels of basal autophagy. When autophagy was inhibited by 3-methyladenine, the VZV titer was markedly reduced. Second, when autophagy was inhibited by Autophagy Related 5 (*ATG5*) siRNA, the VZV titer was reduced. In a third study, they selected the anti-autophagy antibiotic bafilomycin A1 (BAF). BAF is an inhibitor of vacuolar-type H+ ATPase; BAF impairs autophagic flux into the more acidic vesicles. They postulated that BAF would also decrease VZV assembly and infectivity. When BAF was added to infected monolayers midway through the infectious cycle, there was a decline in VZV titers. Studies by confocal microscopy to define a site of a block were inconclusive. However, studies by transmission electron microscopy were striking. After treatment with BAF, numerous naked capsids were seen in both the nucleus and the cytoplasm. However, enveloped particles were not seen in cytoplasmic vacuoles associated with exocytosis nor were virions seen at the plasma membrane. When they reviewed over 600 archived micrographs of control infections, they were unable to find similar examples. In short, BAF specifically blocked secondary envelopment of capsids within the VZV assembly complex in the cytoplasm. No animal or human studies were performed.

Elena Lian (Arthropod-borne and Infectious Diseases Laboratory, Department of Microbiology, Immunology, and Pathology, Colorado State University, Fort Collins, CO, USA) investigated a non-canonical role of arachidonic acid, a pro-inflammatory precursor, as a pro-viral molecule during dengue infection. Dengue viruses (DENVs) pose a severe threat to an estimated 4 billion individuals every year. To better understand the pathogenesis of these viruses, it is critical to determine what host metabolic pathways are deliberately altered to facilitate viral infection as opposed to mediating an antiviral response. One pathway altered upon infection is the arachidonic acid (AA) metabolic pathway, which is canonically antiviral due to its role in inflammation. The function of AA metabolism is ambiguous with regards to DENVs due to the dual roles of AA in lipid metabolism and the immune response. DENVs manipulate lipid metabolism to mediate a massive expansion of the endoplasmic reticulum membrane to replicate; therefore, AA, as a polyunsaturated fatty acid, may be necessary for the membrane architecture required to support viral replication. To identify if AA and the upregulation of its metabolism is truly antiviral during DENV type 2 (DENV2) infection, the synthesis of AA and its transformation into inflammatory products was inhibited: the de novo synthesis of AA was blocked by inhibiting fatty acid desaturase 2, the release of AA from phospholipids was prevented by inhibiting various phospholipase A_2_s, and 5-lipoxygenase was inhibited to block the initiation of AA metabolism. Inhibiting these enzymes decreased infectious viral titer, indicating that AA-related lipid metabolic pathways were critical for DENV2 replication. Therefore, AA metabolism does not play a strictly antiviral role during DENV2 infection but may pose a dual function. No human or animal studies were performed.

Raina Plowright (Montana State University, Bozeman, MT, USA) discussed how the process of pathogen spillover from animals to humans requires a series of events to align in space and time. They explored how non-linear interactions among these events can drive the rate of spillover. Using bat-viruses as a model system, they tracked the virus through the reservoir host population, the environment, and then the human, and examined each barrier that must be overcome to allow cross-species transmission. With data from Hendra virus, they showed how recent ecological changes can determine spillover risk and how this risk can possibly be reversed through strategic ecological interventions. No human or animal studies were performed.

Laura A. St. Clair (Arthropod-borne and Infectious Diseases Laboratory, Department of Microbiology, Immunology and Pathology, Colorado State University, Fort Collins, CO, USA) discussed the relationship between flavivirus infection and sphingolipid metabolism. Sphingolipids have long been recognized for their role in cellular membrane structural integrity. However, sphingolipids have recently emerged as potent, bioactive molecules that mediate several cell-signaling pathways, including the host immune response. Pathogeneses of diseases such as insulin-resistant diabetes, Alzheimer’s, cancer, and viral infection have been linked to perturbations of sphingolipid homeostasis. They and others have previously shown that dengue viruses (DENVs) significantly alter sphingolipid metabolism in both human and mosquito cells and in human sera from infected patients. Inhibition of sphingolipid Δ-4 desaturase (DEGS), involved in the final step of de novo ceramide synthesis, was shown to significantly reduce viral genome replication and infectious particle release in mosquito cells. In sera collected from patients infected with DENVs, a significant increase in sphingolipids during the febrile phase and subsequent down regulation during defervescent and convalescent phases has been observed. Decreases in serum sphingosine-1-phosphate by DENVs has also been shown to be a driving factor of the endothelial barrier failure and subsequent vascular leakage observed in severe dengue disease. These observations provide insight into the immunomodulatory effects of these lipids. They hypothesize that certain metabolites of sphingolipid metabolism are required by DENVs to establish a productive infection, while other metabolites are altered by the host in order to activate the host antiviral response. She presented preliminary data on the mechanisms controlling sphingolipid homeostasis during infection with dengue and Zika viruses, shedding light on virus- versus host-specific arms of the pathway that influence flavivirus disease outcomes. *No human or animal studies were performed*.

### 2.2. Transcription

Joel Baines, along with Claire Birkenheuer (Louisiana State University, Baton Rouge, LA, USA), presented data on the use of Precision Nuclear Run on followed by deep sequencing (PRO-Seq) to determine RNA polymerase II (Pol II) occupancy in cells infected with herpes simplex virus to nucleotide resolution. Within 3 h after infection 30% of Pol II is relocated to viral genes. During this time, Pol II is displaced from cellular promoter proximal pause (PPP) sites and bodies of about 66% of cellular genes, whereas viral infection increased Pol II occupancy on many genes involved in cellular secretion and prevention of apoptosis. Infection caused extension of the termination zone on more than 85% of cellular genes, and this effect correlated with low mRNA accumulation in the cytoplasm. Previous models to explain the production of different viral genes at different times after infection proposed recruitment of Pol II to different temporal classes of herpes simplex virus type 1 (HSV-1) genes at different times. However, they found that Pol II was recruited to all temporal classes of genes by 3 h, despite the longstanding observation that late genes are not efficiently expressed at this time. While recruitment to late genes by 6 h post infection did increase, the most striking difference between immediate early and late genes at early times was efficient movement into immediate early gene bodies, and prolonged pausing of Pol II on late gene PPP sites. In contrast, promoter proximal pausing became prominent on immediate early genes by 6 h post infection, while progression into elongation was rapid on late genes. *De novo* viral protein synthesis was necessary for full Pol II occupancy on all but immediate early genes. Blocking DNA replication with acyclovir and phosphonoacetic acid precluded full Pol II occupancy along the genome, and the remaining Pol II occupied PPP sites, especially on late genes. Collectively, these data suggest that DNA replication is necessary for elongation into the bodies of late genes, and that progression from a paused state to elongation is a major and previously underappreciated contributor to the regulation of HSV transcription during productive infection. No animal or human studies were performed.

Claire H. Birkenheuer, along with Joel Baines (Louisiana State University, Baton Rouge, LA, USA), discussed how transcription of HSV-1 genes is regulated in a cascade-like fashion starting with the expression of immediate early (IE or α) genes and ending with true late genes (γ2). Using cytoplasmic RNA sequencing (RNAseq) precision nuclear run-on (PRO-seq) and global run-on (GRO-seq) they found that at 3 hpi immediate early gene transcription predominates and late genes are poorly transcribed, however, substantial RNA Polymerase II (Pol II) loading was observed on HSV-1 genes of all temporal classes. By 6 hpi, late gene cytoplasmic transcripts are significantly increased, and this is reflected in Pol II occupancy as seen with PRO-seq. However, the most striking difference between genes of different temporal classes was observed by comparing their PRO-seq data with their GRO-seq data at 3hpi. These data showed that Pol II complexes that accumulated just downstream of the transcription start site (TSS) of the IE gene α22 entered the gene body within 5 min. In contrast, Pol II remained paused just downstream of the TSS of the g2 gene UL44 for at least 60 min. From these data, they concluded that the temporal cascade of HSV-1 gene expression is regulated not only by Pol II recruitment onto promoters of different temporal classes, but also by selection of Pol II complexes for elongation at the appropriate time. No human or animal studies were performed.

David Bloom (University of Florida College of Medicine, Gainesville, FL, USA) reviewed the role of HSV’s non-coding RNAs in latency and reactivation. During HSV-1 latency, lytic gene expression is repressed, and abundant RNA expression occurs primarily from the long repeat regions (RLs) of the genome. The HSV-1 latency-associated transcripts (LATs), transcribed from the RLs, have long been considered a hallmark or HSV-1 latency, though it has become clear that only a subset of neurons that contain HSV DNA produce detectable LATs. In addition, at least eight microRNAs (miRNAs), and recently additional long noncoding RNAs (lncRNAs), have been mapped to the LAT region. There are a number of biological phenotypes that have been mapped to the LAT region including functions that facilitate the establishment, maintenance and reactivation from latency as well as facilitating neuronal survival and anti-apoptosis. A central difficulty in dissecting the specific genetic functions responsible for these phenotypes is related to the genetic complexity of the RL region and the tendency of deletion mutants to alter multiple genetic elements. In this talk, he described recent results using ribozymes targeting specific RNAs in vivo using AAV vectors as well as the use of an in vitro human neuronal culture system to dissect LAT and miRNA functions and characterize different profiles of lncRNA expression during latency. All animal studies were performed following guidelines and protocols approved by the Institutional Animal Care and Use Committee of the University of Florida. 

Molly Butler, along with Nunya Chotiwan, Jasmine Donkoh, Connie Brewster, Sandra Quackenbush, Rushika Perera, and Joel Rovnak (Department of Microbiology, Immunology and Pathology, Colorado State University, Fort Collins, CO, USA), shared her work on dengue viruses, among the most aggressive arthropod-borne diseases worldwide, and how these viruses induce metabolic changes within infected cells. Upregulation of metabolic pathways is necessary to meet the demands of viral replication. These metabolic changes, including increased glucose metabolism and autophagy, ultimately promote viral genome replication as well as infectious particle formation and maturation. The mechanisms by which these changes occur are known to be, at least in part, virally directed through complex interactions between host and viral nonstructural proteins. They were interested in the transcriptional regulation of these metabolic changes. They investigated the role of a host transcriptional regulator, cyclin-dependent kinase 8 (CDK8), during dengue infection as a mediator of virally induced metabolic changes. They showed that CDK8 is not only a transcriptional regulator of select metabolic gene expression but also the type I interferon response. This surprising contradiction demonstrates that in the context of dengue infection, CDK8 is a transcriptional regulator of both pro-viral and anti-viral processes. Further, they showed that manipulation of CDK8 activity by chemical inhibition, and CDK8 expression by lentivirus-mediated shRNA knockdown, changes the outcome of dengue infection by influencing viral genome replication and ultimately infectious particle production. CDK8 is therefore a transcriptional hub for induced gene expression during dengue infection and can be manipulated to alter the course of viral infection. No human or animal studies were performed.

Jasmine Donkoh, along with Molly Butler, Connie Brewster, Rushika Perera, Sandra Quackenbush and Joel Rovnak (Department of Microbiology, Immunology and Pathology, Colorado State University, Fort Collins, CO, USA), shared her work regarding Zika virus (ZIKV), a mosquito borne flavivirus that is associated with outbreaks of microcephaly in South America in 2016–2017. ZIKV is linked to cases of Guillain–Barre syndrome, fatal encephalitis and myelitis as well as other neurological complications. DENV, a close relative of ZIKV, has been shown to alter cellular metabolism, resulting in an alteration in metabolites and host proteins. They have found that ZIKV infection leads to induced expression of the host mediator complex protein, cyclin dependent kinase 8 (CDK8) and that CDK8 promotes virus replication. CDK8 is a transcriptional co-factor that regulates gene expression in multiple metabolic pathways. They showed that CDK8, as well as the glycolytic enzyme hexokinase 2 (HK2), are increased during ZIKV infection. Using the CDK8 kinase inhibitor, Senexin A, they demonstrated that CDK8 is required for increased expression of HK2. They also show that ZIKV genomic RNA and particles decrease. The attenuated ZIKV replication may be due to decreased availability of metabolites necessary for nucleic acid and fatty acid synthesis. No human or animal studies were performed.

Sven Heinz, along with Lorane Texari, Michael G.B. Hayes, Max W. Chang, Ninvita Givarkes, Christopher Benner (Department of Medicine, School of Medicine, University of California, San Diego, La Jolla, CA, USA), Lars Pache (Infectious and Inflammatory Disease Center, Sanford Burnham Prebys Medical Discovery Institute, La Jolla, CA, USA) and Matthew Urbanowski, Alexander Rialdi, Kris M. White, Randy A. Albrecht, Ivan Marazzi, Adolfo García-Sastre, Megan L. Shaw (Icahn School of Medicine at Mount Sinai, New York, NY, USA), studied the effects of nuclear replication of influenza A virus (IAV) on host genome 3D structure. They found that IAV disrupts host genome interactions downstream of highly transcribed genes. This effect is caused by IAV non-structural protein 1 (NS1), which inhibits pre-mRNA cleavage at 3′ polyadenylation signals. This results in nuclear retention of unprocessed newly transcribed mRNA transcripts, including of antiviral genes, and failure of RNA polymerase II to terminate transcription. The ensuing read-through transcription past the ends of genes disrupts cohesin-mediated genome interactions downstream, decompacts these regions and frequently switches them from the inactive to the active chromatin compartment. Studies in uninfected cells exposed to strong transcriptional stimuli indicate that RNA polymerase II transcription generally exerts these effects and suggests a mechanism by which transcription contributes to shaping chromosome conformation. All studies using human tissue samples were deemed non-human subject research by the Institutional Review Board of the Icahn School of Medicine at Mount Sinai.

Qing Yang, along with Sara Sawyer and Robin Dowell (Department of Molecular, Cellular and Developmental Biology, BioFrontiers Institute, University of Colorado, Boulder, CO, USA), discussed the potential role of p38 mitogen-activated protein kinase (MAPK) in transcription regulation during type-I interferon response. Type-I Interferon response forms a front line of cell-autonomous defense against viral infection. Until now, the Janus kinase signal transducer and activator of transcription (JAK-STAT) signaling cascade is regarded as the key to initiate expression of interferon-stimulated genes (ISGs), but the underlying transcriptional regulatory network lacks temporal and contextual resolution. Using nascent transcriptional analysis, they identified a group of transcription factors that undergo rapid activation and de-activation in cells undergoing interferon treatment. Through clustering analysis, they revealed that p38 MAPK is the master regulator of these transcription factors. p38 MAPK has been known as the master switch for the nuclear factor kappa-light-chain-enhancer of activated B cells (NF-κB)-mediated inflammatory response. This is achieved via phosphorylation of histone H3 and the transcriptional activator CBP/p300. The p38-mediated phosphorylation creates the necessary chromatin environment for transcription initiation. Surprisingly, inhibition of p38 MAPK also potently suppresses ISG transcription even in the presence of interferon. This strong phenotype suggests that p38 MAPK likely also play an essential role in potentiating ISG transcription. However, competing models suggest that such dependency could also be resulted from p38-dependent STAT1 phosphorylation. Through nascent transcription analysis in the context of p38 inhibition, they hope to resolve the underlying mechanism and add this essential transcription switch to the existing IFN signaling pathway. No animal or human studies were performed.

### 2.3. Pathology

Jayasri Das Sarma (Lerner Research Institute, Cleveland Clinic, Cleveland, OH, USA; Indian Institute of Science Education and Research Kolkata, Mohanpur-741246, India) discussed the protective role of Ifit2, an interferon-induced protein, during neurotropic virus infection. Interferon (IFN) induced tetratricopeptide repeats gene, *ISG54*, represented in mice by IFN induced tetratricopeptide repeats 2 (*Ifit2*) known to be beneficial in protecting mice from lethal neurotropic virus infection by restricting viral replication and amplifying antiviral responses. Using a neurotropic coronavirus infection in *Ifit2* depleted mice they reported that in absence of *Ifit2* viral replication is dramatically increased and mice develop severe neuropathy. Despite the enormous viral load *Ifit2* deficient mice are impaired in microglial activation and recruitment of peripheral innate monocyte/leukocyte into the central nervous system (CNS.) As a consequence, *Ifit2−/−* mice have less encephalitis implied by perivascular cuffing and microglial nodule formation. Homeostatic interaction between microglia, CNS resident neural cells as well as endothelial cells in the blood CNS barrier through chemokines and chemokines receptor known to accelerate efficient leukocyte adhesion and migration into the CNS which may be disrupted in *Ifit2* deficient mice and as a consequence *Ifit2* deficient mice lack protective host innate immunity. C57BL/6 mice and Homozygous *Ifit2* mice on the C57BL/6 background were obtained from the breeding colony of Lerner Research Institute, Cleveland Clinic, OH, and USA. All studies were carried out in strict accordance with all provisions of the Animal Welfare Act, the Guide for the Care and Use of Laboratory Animals, and the PHS Policy on Humane Care and Use of Laboratory Animals. All animal experiments were performed in compliance with protocols approved by the Cleveland Clinic Institutional Animal Care and Use Committee (PHS assurance number A3047-01).

Elizabeth Fortunato (University of Idaho Dept of Biological Sciences, Moscow, ID, USA) shared her work involving the effect of Human Cytomegalovirus on cerebral organoid development. Congenital Human Cytomegalovirus (HCMV) infection causes a broad spectrum of central and peripheral nervous system disorders, ranging from microcephaly to hearing loss. These ramifications mandate the study of host–virus interactions in neural cells. Neural progenitor cells are permissive for lytic infection. They found that more primitive induced pluripotent stem cell (iPSC) lines were susceptible to infection, but not permissive. Differentiation of infected iPSCs induced de novo expression of viral antigens. iPSCs can be cultured in three-dimensions to generate cerebral organoids, closely mimicking in vivo development. Mock- or HCMV-infected iPSCs were subjected to a cerebral organoid generation protocol. HCMV IE1 protein was detected in virus-infected organoids 52 days post-infection. No significant effect on organoid growth was observed, however, infection induced regions of necrosis, large vacuoles, and cysts. Similar to described manifestations of HCMV-induced birth defects, infection dramatically altered neurological development of organoids; decreasing the number of developing and fully-formed cortical structure sites, with associated changes in the architectural organization and depth of lamination within these structures and manifesting aberrant expression of the neural marker b-tubulin III. Their observations parallel published descriptions of infected clinical samples, which often possess sparse antigen positive foci, yet display areas of focal necrosis and cellular loss, delayed maturation, and abnormal cortical lamination. The parallels between pathologies present in clinical specimens and the highly tractable 3D organoid system demonstrate the utility of this system in modeling host–virus interactions and HCMV-induced birth defects. No human or animal studies were performed.

David W. Hawman (Laboratory of Virology, Rocky Mountain Laboratories, NIAID/NIH, Hamilton, MT, USA) shared his work examining the T-cell response of mice to Crimean-Congo Hemorrhagic Fever Virus. Crimean-Congo hemorrhagic fever virus (CCHFV) is a tick-borne virus capable of causing a severe disease within infected humans, Crimean-Congo hemorrhagic fever (CCHF). Cases of CCHF are reported throughout Africa, Eastern Europe, the Middle East and Asia. In addition to bites from infected ticks, humans can become infected with CCHFV during animal husbandry and in the health-care setting during care of infected patients. In humans, CCHF initially presents as a non-specific febrile illness that can then rapidly progress to hemorrhagic manifestations. Case fatality rates can be up to 30%. Despite continued CCHF cases across a wide geographic range, little is known about the host adaptive responses that control the infection. To investigate the host adaptive immune response to CCHFV, they use a mouse model that recapitulates many aspects of severe acute disease and convalescence seen in humans. In this model type I interferon deficient (IFNAR−/−) mice are inoculated with a clinical isolate of CCHFV. Infected mice develop a progressively worsening clinical disease with high viral loads, elevated inflammatory cytokines and significant organ pathology. Recovery from acute disease was coincident with virus specific antibody and activated T-cells. Further studies have shown distinct T-cell responses to CCHFV in the liver and spleen, two key sites of viral replication. Lastly, in vivo studies have shown that depletion of either CD4 or CD8 T-cells results in significantly reduced survival indicating that T-cells are necessary for control of acute CCHFV infection. However, when both CD4 and CD8 T-cells are depleted simultaneously, this exacerbated clinical disease is partially ameliorated, and survival is improved. Cumulatively these data suggest that although T-cells are required for efficient control of CCHFV, unbalanced T-cell responses may be pathogenic. Ongoing studies are further investigating these phenotypes to determine the protective and/or pathogenic role of T-cells in CCHF. All animal studies were performed following guidelines and protocols approved by the Rocky Mountain Laboratories Institutional Animal Care and Use Committee.

Juliette Lewis (Department of Microbiology, Immunology and Pathology, Colorado State University, Fort Collins, CO, USA) shared her work investigating the use of Jamaican Fruit Bats as an animal model to study henipaviruses. Nipah virus (NiV) and Hendra virus (HeV) are pteropid bat-borne viruses in the genus *Henipavirus*—members of which can cause fatal encephalitis in humans and livestock. Recently, another henipavirus, Cedar virus (CedV) was isolated from pteropid bats that is nonpathogenic in all tested animal models. The disparate pathogenicity of CedV and its relatives has yet to be fully investigated, although one hypothesis is that because CedV lacks V and W genes found in NiV and HeV that are potent interferon pathway antagonists. In this study, they tested the susceptibility of epithelial cells from Jamaican fruit bats (*Artibeus jamaicensis*) to these viruses. They also examined antiviral gene expression of the epithelial cells to infection using qPCR arrays. Within two days, conspicuous cytopathic effect (CPE) was present in cultures inoculated with the viruses, including syncytia formation, a common feature of henipavirus infection. By day three, cells inoculated with NiV and HeV exhibited extensive CPE, but this was less pronounced in CedPV inoculated cells. Viral RNA abundance in culture supernatants increased during the study. Expression of several dozen antiviral genes showed that more than half were upregulated, some more than 10,000-fold, in response to CedPV infection, whereas nearly all the genes were repressed during NiV and HeV infection. This work is the first to show that cells from a species of New World bat are susceptible to henipaviruses. CedPV elicited a robust antiviral response that appeared to delay onset of CPE but with viral replication. NiV and HeV repressed the antiviral response and caused substantial CPE and cell death. These results suggest that New World bats may be useful animal models for the study of henipaviruses. All animal studies were performed following guidelines and protocols approved by the Institutional Animal Care and Use Committee of Colorado State University.

Maria A. Nagel (University of Colorado School of Medicine, Aurora, CO, USA) discussed a possible link between varicella zoster virus and Alzheimer’s disease. Alzheimer’s disease (AD) is characterized by accumulation of insoluble forms of amyloid (comprised of Ab42 and amylin) in plaques in extracellular spaces and vessels, aggregation of microtubule protein tau in intracellular neurofibrillary tangles, ischemia and neuroinflammation. The cause of amyloid formation in sporadic AD is complex, but pathogens have been proposed as triggers, interacting with genetic and environmental factors to initiate and/or potentiate disease. Among all the infectious agents that could contribute to AD, varicella zoster virus (VZV) is a strong candidate because, similar to AD, VZV infection produces cerebrovascular disease, neuroinflammation and long-term cognitive impairment. Furthermore, recent epidemiological studies show that VZV reactivation (zoster) significantly increases dementia risk. Thus, they examined how VZV can contribute to AD progression through the production of amyloidogenic peptides and amyloid in cerebrospinal fluid (CSF) from patients with VZV vasculopathy and in VZV-infected primary human perineurial cells and brain vascular adventitial fibroblasts. Compared to stroke controls, VZV vasculopathy CSF had elevated levels of amylin and of amyloid fibrils that correlated with viral titers; Ab40 was significantly reduced and Ab42 unchanged. In VZV-infected cells in vitro, amylin was upregulated and had a proviral function, intracellular amyloid was found, and an extracellular amyloidogenic environment produced. Subsequently, they showed that VZV glycoprotein B peptides form amyloid fibrils and increases fibril formation with amylin and Ab42 in a dose-dependent manner. These results indicate that viruses may contribute to the pathogenesis of AD by upregulating cellular amyloidogenic proteins and producing self-aggregating viral peptides that accelerate amyloid fibrillization. All studies using human subjects or tissue samples have been either approved or deemed non-human subject research by the Institutional Review Board of the University of Colorado.

Lisa K Poppe, along with John T West and Charles Wood (Nebraska Center for Virology and School of Biological Sciences, University of Nebraska, Lincoln, NE, USA), investigated antibody-dependent cellular cytotoxicity (ADCC) as a novel function for Kaposi’s sarcoma-associated herpesvirus (KSHV) antibodies. Kaposi’s sarcoma-associated herpesvirus has been recognized as the etiological agent of Kaposi’s sarcoma (KS) for 25 years; however, little is known about factors that drive disease progression or prevent KS development among those asymptomatically infected. The association of KS with HIV infection, immunosuppression, and high KSHV viral loads suggests an essential role for the immune response in limiting KSHV pathogenesis, but the components responsible remain unknown. For example, T cell responses to KSHV are weak and variable in both KS patients and infected but asymptomatic individuals. In addition, neutralizing antibodies are more frequently detected in individuals with KS. These findings suggest that non-neutralizing antibodies may play a role in preventing KS development. One potential mechanism of action for non-neutralizing antibodies is antibody-dependent cell cytotoxicity; however, no studies have investigated this role for KSHV antibodies to date. Here, they developed and validated an assay to detect ADCC activity of patient plasma against KSHV infected cells. Preliminary data shows ADCC mediating antibodies are not common among asymptomatic individuals and may be positively correlated with antibody titer. Work is ongoing to determine if ADCC responses differ by HIV status or between asymptomatic individuals and patients with KS. All studies using human subjects or tissue samples have been either approved or deemed non-human subject research by the Institutional Review Board of University of Nebraska-Lincoln.

Amber Rico (University of Nebraska-Lincoln, Lincoln, NE, USA) presented a reductionist model for the study of vaccinia B12 and its governing by vaccinia B1. Poxviruses orchestrate complex manipulation of signal transduction to establish productive infection. Vaccinia B1 and B12 form a signal transduction axis recently discovered following adaptive evolution of a B1-deficient virus, ∆B1. This suppressor screen revealed that, in the absence of B1, B12 is catastrophic for viral replication. Here, they developed a reductionist model for B1/B12 signal transduction by expressing B1 and B12 in the absence of other viral proteins. They demonstrated that B12 expressed from a lentiviral vector negatively impacted cellular growth. Importantly, simultaneous expression of B1 from a separate lentiviral vector rescued cellular growth. Immunofluorescence analysis indicated that B12 expression alone resulted in its mis-localization compared to infected cells. Whereas, expression of B12 with B1 resulted in typical nuclear B12 expression. Additional expression and B1-complementation assays confirmed that B1 and B12 co-regulate each other. To study B12 without overt cellular toxicity, they developed an inducible, lentiviral expression system. Inducible B12 inhibited the replication of the adapted virus mutant, ∆B1mutB12, without impacting cellular growth. Together their reductionist models for B12 expression allow for the assessment of B12 toxicity via two readouts, inhibition of cellular growth and viral repression. Ongoing studies are aimed at identifying the mechanism of action for B1/12 regarding poison/antidote activity. Collectively, these results confirm that B1/B12 signal transduction is epistatic in nature with the presence of B12 defining the requirement of B1 and that expression of B12, in the absence of other viral proteins, is sufficient to negatively impact cellular biology and reduce the replication of B1-deficient viruses. No animal or human studies were performed.

Michael J Rudy, along with Penny Clarke and Kenneth Tyler (Neurology Department at the University of Colorado School of Medicine, Aurora, CO, USA), presented his research on Enterovirus D68 (EV-D68) which is an emerging pathogen that causes a biennial respiratory disease and is associated with rapid-onset muscle-weakness and limb paralysis in children (termed acute flaccid myelitis). Recent, but not historic, viral isolates from the United States are capable of infecting spinal cord motor neurons in vivo and neuronal cells in vitro, however the biological basis of the increased virulence and recently acquired ability of contemporary EV-D68 to induce CNS disease is unknown. The presence or absence of an envelope surrounding the capsid of an animal virus influences its stability, transmission, and immune recognition within the CNS. Picornaviruses (including EV-D68) are considered to be “non-enveloped” viruses that package their genomes into icosahedral protein capsids that are released during cell lysis. It has recently been shown that several picornaviruses can be released from cells in a non-lytic fashion via extracellular vesicles. They examined EV-D68 viral structure using ultracentrifugation through a density gradient and found that, in addition to the expected naked viral peak at 1.22 g/cm^3^, there was also a second naked virus peak at 1.19 g/cm^3^ and a third viral peak which was consistent with an enveloped form of the virus (at a density near 1.1 g/cm^3^). Treatment with a non-ionic detergent prior to centrifugation eliminated the “membrane” peak and drove the infectivity into the “naked virus” peaks. In addition, electron microscopy revealed that the membrane contained a conglomerate of viral particles and characterization of nanoparticles via nanosight revealed that these membrane-bound virus particles were approximately 150nm in diameter. Taken together, their data suggests the existence of a previously undiscovered membrane-bound form of EV-D68. No animal or human studies were performed.

Mario Santiago (Department of Medicine, University of Colorado AMC, Aurora, CO, USA) discussed his work examining the differences between IFNα subtypes and IFNβ. Type I interferons (IFN-Is) are cytokines that exhibit potent antiviral activities. Among several IFN-Is, innate immune responses dictated by IFNα and IFNβ are critical roadblocks in the establishment of HIV-1 infection. Notably, IFNα is comprised of 12 distinct subtypes. They previously reported that the clinically-approved subtype, IFNα2, was significantly less potent at inhibiting HIV-1 in primary mucosal CD4+ T cells compared to IFNα6, IFNα8 and IFNα14. The antiviral potencies correlate with the binding affinity to the IFNAR2 subunit of the IFN-I receptor, suggesting that quantitative differences in signaling account for differential antiviral activity. These findings raised the issue of whether the IFNα subtypes and IFNβ have qualitative differences that may have driven their evolution. To address this controversy, they titrated recombinant IFNα subtypes and IFNβ in a reporter cell line harboring the IFN response element (ISRE) of a canonical IFN stimulated gene, ISG15, linked to luciferase. As expected, triggering of the ISRE-luciferase cell line correlated significantly with IFNAR2 binding affinity, indicating that this assay measures quantitative differences in IFNAR signaling. Dominant IFNα subtypes (IFNα1, IFNα2, IFNα5, IFNα8 and IFNα14) expressed in HIV-exposed plasmacytoid dendritic cells and IFNβ were normalized for quantitative differences then incubated with purified mucosal CD4+ T cells (*n* = 3 donors) for 18 h. The corresponding “interferomes” were evaluated by RNAseq, with differentially-induced genes set at a threshold of 1.5-fold relative to mock. If there were no qualitative differences, they expected substantial overlap of the interferomes. Notably, they observed the following: (1) IFNβ altered up to 3-fold more genes compared to individual IFNα subtypes; (2) the overlap of interferomes between the IFNα subtypes ranged from 59% to 82%; (3) IFNα14 altered 243 genes that were not altered by other IFNα subtypes; and (4) IFNα5 downregulated multiple genes that were upregulated by the other IFNα subtypes tested. Ingenuity Pathway Analyses suggested processes that could be regulated by some IFN-Is but not others. Overall, their data provide strong evidence that distinct IFN-Is have qualitative differences that could have important implications in harnessing these cytokines for antiviral control. All studies using human subjects or tissue samples have been either approved or deemed non-human subject research by the Institutional Review Board of the University of Colorado.

Nicole R. Sexton, along with Eric D. Bellis, Mark Cole Spangler, Reyes M. Murrieta, James Weger-Lucarelli and Gregory D. Ebel (Department of Microbiology, Immunology and Pathology, Colorado State University, Fort Collins, CO, USA), investigated the composition of Zika Virus infectious units, demonstrating that single cell infections likely begin with multiple genomes. Zika virus (ZIKV, *Flaviviridae, Flavivirus*) is an arthropod-borne infection that can result in severe outcomes, particularly on fetuses infected in utero. Exactly how ZIKV is transmitted from cell-to-cell or from mosquito-to-human is unknown. It has been assumed that infection by ZIKV, as well as other viruses, is largely initiated by individual virus particles binding and entering a cell. However, recent works have demonstrated that viruses can deliver multiple virus particles simultaneously, and that this collective particle delivery enhances infection. ZIKV transmits cyclically between *Aedes aegypti* mosquitos and vertebrate hosts, including humans. Human infection is initiated through the injection of a relatively small initial inoculum comprised of a genetically complex virus population. Since most mutations decease virus fitness, collective particle transmission could benefit ZIKV and other arthropod-borne diseases by facilitating the maintenance of genetic complexity and adaptability during infection, as well as through other mechanisms. Here they utilized a barcoded ZIKV to infect African green monkey cells and picked individual plaques for sequencing to determine the number of virus genomes that initiated each plaque. They found a range of one to 212 virus genomes initiated individual plaques (average of 10), but very few results in more than two dominant genomes. Populations of infectious units of ZIKV were separated mechanically by centrifugation and larger infectious units were found to reform after removal. Overall, these data suggest that ZIKV populations are made up of a variety of infectious unit sizes and only rarely begin with a single virus genome. No animal or human studies were performed.

Hadrian Sparks (Department of Immunology and Microbiology University of Colorado School of Medicine, Aurora, CO, USA) discussed how manipulation of flavivirus 3′ untranslated region (UTR) structural interactions may be used as a novel vaccine attenuation strategy. Similar to other mosquito-borne flaviviruses, Zika virus (ZIKV) encodes two RNA structures (xrRNA1 and xrRNA2) in the 3′ untranslated region (UTR) of the viral genome that prevent degradation by host 5′-3′ exoribonuclease 1 (Xrn1). Viral RNA-mediated Xrn1 resistance results in sub-genomic flaviviral RNAs (sfRNAs) from the undigested 3′ UTR which accumulate during infection of the host cell. Previous studies have shown that xrRNA-dependent sfRNA production is necessary for replication and pathogenicity of flaviviruses. However, little is understood about the function of ZIKV xrRNA structural elements in sfRNA production, viral replication, and host–virus interactions. Recently, the ZIKV xrRNA1 tertiary structure was solved and used to predict novel interactions within the xrRNA1 pseudoknot that are essential for resistance of host Xrn1 activity. A single cytosine at position 22 of xrRNA1 is thought to stabilize the phosphate backbone kink that forms the RNA pseudoknot. Based on this information, they hypothesize that replacing the cytosine with a guanine base at this position in either Zika xrRNA1 or xrRNA2 will decrease the production of sfRNA during infection leading to reduced viral replication and pathogenicity. They generated infectious ZIKV clones with a C22G mutation in xrRNA1 or a C103G mutation in xrRNA2. They showed that xrRNA1 and xrRNA2 mutants do not exhibit decreased viral replication during infection when compared to wild type (WT), clone-derived ZIKV in both mosquito and mammalian cell lines. However, they have found that these xrRNA mutants produce an attenuated infection phenotype when used to infect a well-established in vivo mouse model. All animal studies were performed following guidelines and protocols approved by the Institutional Animal Care and Use Committee of the University of Colorado Anschutz Medical Campus.

Joseph Timpona (BioFrontiers Institute, University of Colorado Boulder, Boulder, CO, USA) discussed his work examining the potential role of pathogens in driving the evolution of the host complement system. The complement system comprises a suite of circulating immunity proteins that detect and destroy pathogens. Consequently, complement proteins are targeted by many pathogens—either for evading complement-mediated degradation or for co-opting to promote infection. They analyzed the 56 annotated complement genes for patterns of positive selection in simian primates and revealed that more than half of complement genes contain rapidly evolving codons. To uncover potential host-pathogen interfaces driving rapid evolution, they assessed co-crystal structures of complement proteins in complex with virus proteins. Using the co-crystal structure of echo six virus capsid with its cellular attachment receptor, decay accelerating factor (DAF), they found that key amino acids facilitating their interaction are rapidly evolving in primates. As such, echoviruses may be driving the rapid evolution of DAF in primates, and DAF in turn may serve as a species barrier to echovirus spillover events. No human or animal studies were performed.

Cody Warren (BioFrontiers Institute, Department of Molecular, Cellular, and Developmental Biology, University of Colorado Boulder, Boulder, CO, USA) presented work on the selective use of primate CD4 receptors by HIV-1. Individuals chronically infected with HIV-1 harbor complex viral populations within their bloodstreams. New infections are established by only one or a few virions from within this complex viral swarm. An important goal is to characterize the biological properties of HIV-1 virions that seed and exist early in new human infections because these are potentially the only viruses against which a prophylactic HIV-1 vaccine would need to elicit protection. This includes understanding how the Envelope (Env) protein of these virions interacts with the T- cell receptor CD4, which supports attachment and entry of HIV-1 into target cells. They examined early HIV-1 isolates for their ability to infect cells via the CD4 receptor of 15 different primate species. They found that most primary isolates of HIV-1 from the blood, including early isolates, are highly selective and enter cells through some primate CD4 receptor orthologs but not others. They showed that the weak CD4 binding affinity of most blood-derived HIV-1 isolates is what makes them sensitive to the small sequence differences in CD4 from one primate species to the next. Since weak CD4 binding is nearly universal property of HIV-1 circulating in the bloodstream, it must be a selected and important property in the biology of HIV-1 in the body. They identified six primate species that encode CD4 receptors that fully support the entry of early HIV-1 isolates despite their weak binding affinity for CD4. These findings will help inform long-standing efforts to model HIV-1 transmission and early disease in primates. No human or animal studies were performed.

Joseph A. Westrich (Colorado State University, Fort Collins, CO, USA) presented work examining the effect of the maternal immune response to ZIKV on fetal development. Adverse fetal outcomes occur in roughly 2%–4% of all live births—of which, 7%–10% are due to preventable environmental factors including infections. In addition to the detrimental effect of the pathogen itself, the maternal immune response to the pathogen inadvertently contributes to fetal outcomes. Immune responses at the maternal–fetal interface have been shown to be detrimental for the developing fetus. How the peripheral maternal and fetal viral infections impact the immunological environment at the maternal–fetal interface remains poorly understood. To understand how the antiviral immune responses to these insults effect fetal development, they have established models of maternal antiviral responses in both guinea pigs and mice. Animals during mid gestation were exposed to either Zika virus (ZIKV) or poly I:C, to mimic fetal and peripheral infections, respectively. ZIKV has been shown to cross the placental barrier and negatively affect fetal development during both clinical and subclinical infections. Alternatively, poly I:C, a non-infectious viral analogue, was used to invoke a peripheral antiviral immune response. They have evaluated both the immunological landscape of the maternal–fetal interface and its effect on growth factors known to be altered during adverse fetal development. Their project aim is to understand what immune and growth factors lead to adverse fetal development instigated by the antiviral immune response. As prevention of viral infections during pregnancy is impractical, these findings would allow for a greater understanding of their impact on fetal health and to develop therapeutic interventions that may lessen the impact of these infections. All animal studies were performed following guidelines and protocols approved by the Institutional Animal Care and Use Committee of Colorado State University.

### 2.4. Epidemiology

Sabra L. Klein (Johns Hopkins Bloomberg School of Public Health, Baltimore, MD, USA) presented data on the effects of biological sex on adaptive immune responses, and how those effects may impact vaccine efficacy. To test whether greater antibody in females is sufficient for protection against influenza, males and females were immunized with an inactivated H1N1 vaccine that induced predominantly antibody-mediated immunity. Following vaccination, females had greater antibody responses and protection against challenge with an H1N1 drift variant virus than males. Antibody derived from vaccinated females was better at protecting both naïve males and females than antibody from males, and this protection was associated with increased antibody specificity and avidity to the H1N1 virus. The expression of *Tlr*7 was greater in B cells from vaccinated females than males and was associated with reduced DNA methylation in the *Tlr*7 promoter region, higher neutralizing antibody, class switch recombination, and antibody avidity in females. Deletion of *Tlr*7 reduced sex differences in vaccine-induced antibody responses and protection following challenge and had a greater impact on responses in females than males. Sex differences in vaccine efficacy diminished with age in mice. To determine the role of sex steroids in vaccine-induced immune responses, adult mice were gonadectomized and hormones (estradiol in females and testosterone in males) were replaced in subsets of animals before vaccination. Vaccine-induced antibody responses were increased in females by estradiol and decreased in males by testosterone. The benefit of elevated estradiol on antibody responses and protection against influenza in females is diminished with age in both mice and humans. Taken together, both sex steroids and X-linked genes affect antibody production and results in sex-specific differences in the efficacy of vaccination against influenza. This work was supported by the NIH/NIAID Center of Excellence in Influenza Research and Surveillance contract HHS N272201400007C and the NIH/ORWH/NIA Specialized Center of Research Excellence in Sex Differences U54AG062333. All animal studies were performed following guidelines and protocols approved by the Institutional Animal Care and Use Committee of John Hopkins Bloomberg School of Public Health.

Sara R. Privatt (Nebraska Center for Virology and School of Biological Sciences, University of Nebraska, Lincoln, NE, USA) discussed a longitudinal quantification of adenovirus neutralizing responses in Zambian mother–infant pairs in order to determine the efficacy of adenovirus vaccine strategies. Vaccination offers the most cost-effective approach to limiting infectious and neoplastic diseases that reduce the quality of life in sub-Saharan Africa (SSA). It is unclear what vaccine vectors would be most implementable in the setting and at what age they should be applied for maximal efficacy. Adenoviruses (Ads) and Ad-based vectors have been demonstrated to induce effective humoral and cellular immune responses in animal models and in humans. However, because immunity associated with Ad infection is lifelong, there exists a debate as to whether pre-existing immunity might decrease the efficacy of Ad vectored vaccines. To begin developing vaccination strategies for SSA, they have quantified neutralizing antibodies (nAb) against Ad4, Ad5, Ad7, Ad26, Ad28, Ad45 and Ad48 in 67 adult women and their infants. They are the first to define the decay kinetics of transferred maternal nAb in infants as well as the initiation of de novo Ad responses. Their findings demonstrate that in Zambian adults, robust nAb responses exist against each of the Ads tested and are efficiently transferred to newborns. Neither the HIV-1 infection status of the mothers or the antiretroviral therapy (ART) treatment of HIV-1 had significant impact on maternal Ad nAb responses or their transfer to infants. Maternal Ad nAb decays in infants to a nadir at 12 months of age such that any of the seven Ad types could function as vaccine vectors. The definition of this “window of opportunity” provides important foundational data for rational design and implementation of Ad vectors in this setting. All studies using human subjects or tissue samples have been either approved or deemed non-human subject research by the Institutional Review Board of the University of Nebraska-Lincoln and the University Teaching Hospital in Lusaka, Zambia.

Sydney Townsend, along with Catherine Chunda-Liyoka, John T. West, and Charles Wood (Nebraska Center for Virology, University of Nebraska-Lincoln, Lincoln, NE, USA; Department of Pediatrics and Child Health, University Teaching Hospital, Lusaka, Zambia), detected high NNRTI resistance levels in HIV-1 infected Zambian mothers and infants. In 2015, Option B+ was implemented in sub-Saharan Africa in an effort to prevent mother to child transmission (PMTCT) of HIV-1. Option B+ involves treating pregnant and lactating women with combination antiretroviral therapy (ART) for life and giving the infant prophylaxis immediately post-delivery. Option B+ has been successful in reducing the MTCT rate, but the lack of resources in Zambia has left many mothers/infants susceptible to drug resistance. Drug resistance mutations (DRM) can develop quickly under selection pressure and may linger for long periods of time. Thus, it is important to elucidate relationships between ART resistance in mothers and their HIV-1 infected infants, to investigate if drug resistant variants are transmitted from mother to infant and to quantify prevalence of drug resistance post-Option B+ implementation. Dried blood spots (DBSs) were collected from mother–infant pairs during routine HIV-1 screens in Lusaka, Zambia from 2015 to 2018, DNA was extracted, and HIV *pol* was amplified, sequenced, and submitted to Stanford HIVdb (https://hivdb.stanford.edu/). Forty-nine percent of samples had at least one DRM and were resistant to at least two ARTs. Infants had greater amounts of DRM leading to higher magnitude resistance, and the prevalence of NNRTI resistance was significantly higher than that of other ART classes. Further studies on how to reduce the prevalence of DRMs in infants are needed, and they emphasize the necessary change to protease inhibitor (PI)-based and integrase inhibitor (INI)-based regimens for treatment of HIV-1 infected infants and mothers in Zambia. All studies using human subjects or tissue samples have been either approved or deemed non-human subject research by the Institutional Review Board of the University of Nebraska-Lincoln.

Sue VandeWoude (Department of Microbiology, Immunology and Pathology, Colorado State University, Fort Collins, CO, USA) presented data generated by her laboratory and colleagues from University of Minnesota, and wildlife agencies in Florida, Colorado and California relating to emerging viral outbreaks resulting from host switching. Free-ranging Puma concolor (puma) have been infected with 5 contact-dependent agents originating in domestic cats and bobcats, presumably via predator–prey interactions that overwhelmingly favor survival of the puma. More than 50 individual cross-species transmission events inferred from feline retroviral genomic data, including: (1) spillover of feline immunodeficiency virus infection of bobcats (FIVlru) to pumas, (2) feline leukemia virus (FeLV) and (3) feline foamy virus (FFV) from domestic cats to puma. Interestingly, each of these infections has a different outcome in the new host. FIVlru has high fitness in the bobcat but replicates poorly and is under positive selection in the puma. Feline Leukemia Virus from domestic cats readily causes disease in pumas, and domestic cat FeLV replicates faster in puma versus domestic cat cells. FFV spreads efficiently from puma to puma, with limited host restriction barriers for onward transmission. A series of complex parameters ranging in scale from puma habitat use to intrinsic molecular interactions between virus and ‘new host’ determine risk and outcome of these cross-species infections. All animal studies were performed following guidelines and protocols approved by the Colorado State University Institutional Animal Care and Use Committee.

### 2.5. Diagnostic and Therapeutic

Shidizja Altamirano (Department of Microbiology, Immunology and Pathology, Colorado State University, Fort Collins, CO, USA) discussed paratransgenic delivery of dsRNA to stimulate the mosquito anti-viral RNAi response against a recombinant Sindbis virus containing enhanced green fluorescent protein (Sindbis-eGFP). The Yellow Fever mosquito, *Aedes aegypti*, is one of the most prevalent vectors of human diseases and is known to transmit, in addition to yellow fever, chikungunya, dengue, and Zika viruses. These arboviral diseases affect the health of people inhabiting tropical and subtropical regions and are growing contributors to global morbidity and mortality. As such, efforts to manage mosquito populations and the spread of the pathogens they vector are of great importance to improve the health of humans world-wide. In this project, transgenic bacterial symbionts have been developed, with the goal of introducing ds-eGFP to the mosquito midgut for subsequent interruption of arbovirus infection in the recombinant Sindbis-eGFP (Togaviridae) virus system. The dsRNA is expected to serve as a trigger of the mosquito’s RNA interference anti-viral immune pathway. Their hypothesis is that, once inside of the mosquito midgut, ds-eGFP (~430 nts) is processed by the mosquito RNAi immune pathway. In this project, they developed a quantitative assay for detection of those sRNAs in mosquito midguts. Prevalence of the bacteria-transcribed, mosquito-processed sRNAs were determined using qPCR and standardized to the number of colony forming units on Luria broth agar plates. This project will help us to identify specific symbiont strains that colonize mosquito midguts most efficiently and release ds-eGFP for processing into sRNA RNAi triggers. With additional research and testing, this method could provide vital information for optimization of paratransgenic bacteria strains. No human or animal studies were performed.

Laura Ashton, along with Jasmine McCoy, Garin Wilson, Mariah Jordan, Sandra Quackenbush, and Amy MacNeill (Department of Microbiology, Immunology and Pathology, Colorado State University, Fort Collins, CO, USA), showed that MYXVorfC, a myxoma virus that expresses a pro-apoptotic protein, induces oncolysis through apoptosis and is attenuated in rabbits. Oncolytic virotherapy using myxoma virus (MYXV) has shown efficacy in some cancer models. However, MYXV replication within tumors is short-lived and successful treatment requires frequent injections. It was hypothesized that engineering a recombinant MYXV that promotes apoptosis (MYXVorfC) could improve the efficacy of MYXV. A significant increase (*p* < 0.05) in cell death due to apoptosis was observed in MYXVorfC as compared to MYXV-infected cells 48 h post-inoculation (hpi) in rabbit kidney epithelial, canine osteosarcoma, and canine soft tissue sarcoma cells. Activation of cytochrome C oxidase was significantly (*p* < 0.05) increased in MYXVorfC as compared to MYXV or mock-infected cells by 8 hpi. To assess the safety of MYXVorfC in vivo, New Zealand white rabbits were challenged with MYXVorfC or wild-type MYXV. Rabbits infected with MYXVorfC survived longer (*p* < 0.05) and had a delayed onset of clinical signs as compared to rabbits infected with MYXV. Future studies will determine if MYXVorfC is a more effective oncolytic virus than MYXV. *Funding: College of Veterinary Medicine and Biomedical Sciences and The Animal Health Innovation Fund. “Evaluation of pathogenesis of MYXVorfC in rabbits.* All animal studies were performed following guidelines and protocols approved by the Institutional Animal Care and Use Committee of Colorado State University.

Nathaniel D. Denkers (Prion Research Center, Department of Microbiology, Immunology, and Pathology, Colorado State University, Fort Collins, CO, USA) presented a comparison of real-time conversion and immunohistochemistry techniques for detection of chronic wasting disease in white-tailed deer. Chronic wasting disease (CWD) continues to expand across North America and Canada, and more recently was discovered in Scandinavia. While the exact mechanism of CWD transmission has yet to be elucidated, early diagnosis of infected animals remains a priority in curtailing its spread. While immunohistochemistry (IHC) remains the gold standard for diagnosing CWD, real-time quaking induced conversion (RT-QuIC) can detect substantially lower concentrations of prions. Here they sought to compare the sensitivity of RT-QuIC and IHC on a series of longitudinal tonsil and recto-anal mucosal lymphoid tissue (RAMALT) biopsies from CWD inoculated white-tailed deer throughout disease progression. RT-QuIC detected seeding activity in tonsil biopsies on average of 2.2 months (3–12 months) earlier than IHC detection, and on average of 4.1 months (3–15 months) earlier in RAMALT biopsies. Detection was concurrent by both methods in all remaining biopsies and IHC positive results never preceded a positive RT-QuIC result. Additionally, for biopsy samples that were already positive by both RT-QuIC and IHC at a previous collection, RT-QuIC detected seeding activity in follow-up biopsies where no lymphoid follicles were present to confirm with IHC. Biopsies from CWD(−) deer were negative throughout the study. Overall, these results demonstrate that RT-QuIC detected CWD positivity in both tonsil and RAMALT biopsies approximately 2–4 months prior to IHC. Moreover, seeding activity was detectable even when lymphoid follicles were not present to permit meaningful IHC analysis. These data indicate that RT-QuIC is more sensitive than IHC in detecting CWD infection and could be useful in implementing management strategies. All animal studies were performed following guidelines and protocols approved by the Institutional Animal Care and Use Committee at Colorado State University.

Caitlyn Kraft (Prion Research Center, Department of Microbiology, Immunology and Pathology, College of Veterinary Medicine and Biomedical Sciences, Colorado State University, Fort Collins, CO, USA) presented her research on detection of chronic wasting disease prions in nasal swab collections from CWD-inoculated deer. Chronic wasting disease (CWD) is an emergent transmissible spongiform encephalopathy of cervids now identified in North America, South Korea, and Scandinavia. The exact mechanisms of CWD transmission remain unknown; thus, efforts to identify the pathways of prion shedding and transmission remain a high priority. Previous studies have shown that nasal olfactory epithelium can contain CWD seeding activity, yet the temporal profile of prion shedding in nasal secretions has not been identified. In this study, they characterize CWD pathogenesis using longitudinal nasal swab samples from twenty (20) CWD-inoculated deer by iron-oxide magnetic extraction coupled with real-time quaking induced conversion (IOME-RT-QuIC) protocol. They correlated these assay findings with those from longitudinal tonsil biopsies and terminal tissue samples from the same deer assayed by immunohistochemistry and RT-QuIC. Their results demonstrate prion shedding in nasal secretions in 11 of 20 deer as early as two months after first tonsil biopsy positivity (mean = 10.3 months; range: 2–17 months). Once detected, persistent prion shedding in nasal fluid occurred of 9 of the 11 deer. Prion seeding activity in frontal cortex and olfactory bulb regions of the brain was demonstrated in 15 of 20 deer, suggesting that brain involvement precedes nasal shedding. They conclude that CWD-infected deer shed prions in nasal secretions throughout mid to late stages of pathogenesis. In that direct nasal contact is common in social interactions, it is likely that nasal fluids play a significant role in the horizontal transmission. Moreover, nasal swabs may also constitute an accessible sample for antemortem diagnosis using the RT-QuIC assay. All animal studies were performed following guidelines and protocols approved by the Institutional Animal Care and Use Committee of Colorado State University.

W. Ian Lipkin (Center for Infection and Immunity, Mailman School of Public Health, Columbia University, NYC, NY, USA) gave a post-prandial tour reviewing the promise of new tools for microbial discovery and diagnostics, the opportunities that younger members of the audience will have to expand the field of microbiology into chronic as well as acute diseases, the pitfalls of sensitive molecular methods for pathogen discovery, and ways in which microbiology can help us understand disruptions in the social fabric that pose a pandemic threat at least as real as Ebola or influenza. No human or animal studies were performed.

Amy MacNeill, along with Laura Ashton (Department of Microbiology, Immunology and Pathology, Colorado State University, Fort Collins, CO, USA), demonstrated that myxoma virus (MYXV) replication in neoplastic canine cells is affected by type 1 interferons (IFNs). A better understanding of the immunological response of canine cells against MYXV is needed to improve the effectiveness of myxoma virus (MYXV) as an oncolytic therapy in dogs. The cytokine responses to MYXV inoculation of cancer cells (STS and OSA) permissive to MYXV infection were compared to non-permissive canine fibroblasts. Using qPCR, cytokine mRNA expression in MYXV-infected cells were calculated relative to mock-infected cells at 4 and 24 h post-inoculation (hpi). IFN-α transcription was significantly (*p* < 0.05) lower in STS 4 hpi and OSA 24 hpi. IFN-γ was upregulated 4 hpi in OSA and 24 hpi in STS. Changes in IL-1β, IL-4, and IFN-β transcription were inconsistent between cancer cells. Previous work with human and murine cancer cells indicate that species-specific combinations of cytokines can block MYXV infection. Therefore, they treated STS and OSA cells with TNFα, IFN-α, IFN-β, and IFN-γ alone and in combinations. Treatments were not cytotoxic to STS or OSA cells. Significant (*p* < 0.05) reductions in viral replication were observed in canine cancer cells treated with IFN-α alone or in combination. They propose that regulation of type I IFN responses could allow for targeted adjustments to MYXV replication in spontaneous tumors of dogs. Funding: Colorado State University Clinical Pathology Research and Development Funds. No animal or human studies were performed.

Nathan Mankovich advised by Michael Kirby (Department of Mathematics, Colorado State University, Fort Collins, CO, USA) presented mathematical techniques to accurately detect whether a human subject is shedding the influenza virus using only a small number of gene expression levels. These methods consist of multiple network-based feature selection algorithms for detecting these discriminative biomarkers. These methods both begin with realizing a microarray data set as a network using correlation, partial correlation, heat kernel and random edge generation methods. Then a small set of genes are selected from these networks via the spectrum of the graph Laplacian, adjacency, and supra-adjacency matrices. *No human or animal studies were performed as these methods were used on the GSE 73072 dataset available on NCBI website.*

Erin McNulty (Colorado State University, Fort Collins, CO, USA) discussed how prions are highly resistant to inactivation by conventional sterilization processes. The Center for Disease Control (CDC) recommendation for decontamination of the infectious agent associated with prions include exposure to 20,000 parts per million (ppm) sodium hypochlorite (30–60 min), or high pressure-autoclave (134C for 1 h). High pressure-high temperature autoclaving is typically used to inactivate prions present on plastics, personal protective equipment and within small quantity tissues/homogenates routinely used by laboratory personnel. Laboratory benches, equipment and instruments are routinely exposed to 20,000 ppm sodium hypochlorite. Due to the corrosive nature of high sodium hypochlorite concentrations investigators have searched for effective means to inactivate prions that will result in less damage to the laboratory setting. To this end 4% acetic acid sodium lauryl sulfate (4% acetic SDS) has been incorporated into prion laboratory cleaning regimens. In this study they explored the efficacy of short-term exposure to 20,000 ppm hypochlorite or 4% acetic SDS to inactivate prion infectivity in a laboratory setting by use of prion amplification and mouse bio-assays. They demonstrated that a laboratory cleaning regimen including short-term exposure to 20,000 ppm hypochlorite results in sterilizing inactivation of CWD prion infectivity while 4% acetic SDS decreased CWD burden by 17%. This study provides findings to guide development of prion laboratory cleaning procedures. All animal studies were performed following guidelines and protocols approved by the Institutional Animal Care and Use Committee of Colorado State University.

Amy Nalls (Colorado State University, Fort Collins, CO, USA) discussed detection of hematogenous prions in Chronic Wasting Disease studies. Blood contains the infectious agent associated with prion disease affecting several mammalian species, including humans (Creutzfeldt-Jakob disease (CJD)), cervids (chronic wasting disease (CWD)), sheep (scrapie), and cattle (bovine spongiform encephalopathy (BSE)). It has been confirmed that sufficient prion agent is present in the blood of both symptomatic and asymptomatic carriers to initiate the amyloid templating and accumulation process that results in these fatal neurodegenerative diseases. Yet, to date, the ability to detect blood-borne prions by in vitro methods remains difficult. Their work has focused on refinement of the prion amplification methods RT-QuIC and PMCA. They have utilized banked blood samples collected from CWD studies in the native white-tailed deer host to examine hematogenous prion load in blood collected from deer orally-inoculated with various amounts and sources of CWD. They demonstrated prion seeding activity in buffy coat cells harvested from subclinical and clinical CWD-positive deer, the ability to detect prions in buffy coat blood cells harvested from deer orally dosed with 300 ng CWD-positive brain or saliva, and detection of prions in as few as 5 × 10^5^ buffy coat cells harvested from subclinical CWD-positive deer. These findings permit assessment of the role hematogenous prions play in the pathogenesis of CWD and provide tools to assess the same for prion diseases of other mammalian species. All animal studies were performed following guidelines and protocols approved by the Institutional Animal Care and Use Committee of Colorado State University.

Trey K. Snell (Department of Microbiology, Immunology and Pathology, Colorado State University^,^ Fort Collins, CO, USA) presented his work in developing immunofluorescence and immunoprecipitation assays for characterization of paratransgenic delivery of dsRNA to mosquito midguts. The *Aedes aegypti* mosquito plays a major role as an arbovirus transmission vector. In this project, transgenic bacterial symbionts have been developed, for the purpose of introducing ds-eGFP to mosquito midguts for subsequent interruption of arbovirus infection using the recombinant Sindbis-eGFP (Togaviridae) virus system. They expect that bacterial-delivered dsRNA will serve as a trigger of the mosquito’s RNA interference (RNAi) anti-viral pathway and reduce viral replication. Here, confocal microscopy was used to demonstrate that ds-eGFP was delivered to mosquito midguts. In addition, immunoprecipitations of an RNA-induced Silencing Complex (RISC) protein (Ago2-FLAG) were performed to show that dsRNA was processed into sRNAs by the RISC. Different strains of *Escherichia coli (E.* coli, HT27), and symbionts were transformed to express *ds-GFP* and/or Ago2-FLAG. Bacteria were introduced to adult mosquitoes using a sucrose feeding solution. Four days later, midguts were harvested for confocal microscopy. In a second experiment, Ago2-FLAG/sRNA complexes were immunoprecipitated from mosquito homogenates. Subsequent RNA extractions were used to isolate sRNAs and RT-qPCR of sRNAs was performed. These investigations will help us build support for their hypothesis that paratransgenic delivery of RNAi triggers produces functional anti-viral sRNAs in mosquitoes that can reduce arboviral loads. No human or animal studies were performed.

